# Preoperative and Surgical Predictors of the Treatment Outcome of Dairy Cows with Right Abomasal Displacement—A Retrospective Study of 234 Cases

**DOI:** 10.3390/ani13182887

**Published:** 2023-09-11

**Authors:** Ioannis Proios, Walter Grünberg

**Affiliations:** 1 Clinic for Cattle, University of Veterinary Medicine Hannover, Foundation, Bischofsholer Damm 15, D-30173 Hannover, Germany; 2 Clinic for Ruminants and Herd Health Management, Faculty of Veterinary Medicine, Justus-Liebig-University Giessen, Frankfurter Str. 104, D-35392 Giessen, Germany; walter.gruenberg@vetmed.uni-giessen.de

**Keywords:** cattle, indicator, laparotomy, omentopexy, outcome, prognosis, survival, volvulus

## Abstract

**Simple Summary:**

The right displacement of the abomasum (RDA) is a common and potentially life-threatening disease of dairy cows. Affected cows require early diagnosis and timely surgical treatment. Cows at increased risk of developing complications and a possibly fatal outcome must be identified early in order to guide medical and economic decision making. In this retrospective study, the laboratory and clinical parameters of cows with confirmed diagnoses that were admitted and treated at a veterinary teaching hospital were studied. Treated cows were either classified as survivors (discharged alive) or non-survivors (died or euthanized during hospitalization), and investigated parameters were compared between groups. Cows that died or had to be euthanized showed more pronounced dehydration and increase in heart rate. Distensions of the bowels were more pronounced, and complicated twists of the forestomaches and stomach were more frequent in non-survivors than survivors. Cows unable to stand at the time of surgery all had a fatal outcome. A number of blood biochemical parameters were found to be associated with the treatment outcome and could therefore assist the early identification of cows requiring more intense peri- and postoperative care.

**Abstract:**

The displacement of the abomasum to the right (RDA) is a common condition regularly encountered in dairy cows, which requires urgent surgical correction. The survival of the patient primarily depends on early diagnosis and timely treatment, but other factors contributing to the outcome have been discussed in the literature. The objective of this study was to identify preoperative clinical, hematological, as well as intraoperative parameters that are associated with the prognosis of cows with RDA or abomasal volvulus (AV). This retrospective study included patients admitted to a veterinary teaching hospital over a period of 6 years with a diagnosis of RDA or AV. A total of 234 cows were included, of which 193 were discharged after treatment and thus classified as survivors. In contrast, 41 cases died or were euthanized during or after surgery and were categorized as non-survivors. Non-survivors showed more severe dehydration, higher heart rate, lower sodium, as well as higher L-lactate and phosphorus concentration in their blood prior to surgery compared with the survivors. During surgery, the abomasum of non-survivors was markedly dilated and twisted more frequently than in survivors. The results presented here can facilitate the early identification of animals with poor prognosis requiring more intensive peri- and postoperative care.

## 1. Introduction

Abomasal displacement either to the left or to the right is a common condition of dairy cows frequently encountered in herds with suboptimal transition cow management [1] and, in general, requires surgical intervention [2]. Although abomasal displacement to the left (LDA), has a substantially higher incidence rate than the displacement to the right (RDA), it is this latter form that is associated with higher complication rates and lower survival rates [3]. Because of the risk of volvulus of the abomasum when the abomasum is displaced to right, a patient with this diagnosis is considered a medical emergency. In the case of an abomasal volvulus (AV), severe and sustained strangulation of the organ can lead to impaired vascular perfusion with pronounced and potentially irreversible tissue damage [4]. Clinical differentiation between RDA and AV during a clinical examination can be challenging, even ultrasonographically. The diagnosis of AV thus generally requires confirmation during surgery [5].

The earliest studies investigating parameters suitable for predicting the outcome of cows with RDA or AV were published over 40 years ago [6]. These studies reported poor prognosis to be associated with clinical findings such tachycardia or dehydration [7,8], as well as with blood biochemical parameters such as blood base excess [9,10], serum potassium [7,8,11], serum chloride [8,9,10,12], sodium [2,10], anion gap [8,10], and L-lactate in serum [12,13,14,15].

Apart from laboratory values, clinical parameters determined intraoperatively like the presence and the grade of volvulus and the color of the abomasal wall were suggested as prognostic indicators [4]. Some of these potential predictors were previously investigated [2,8,10,16]. Improving the reliability of a prognosis of the treatment outcome in patients with RDA or AV remains challenging and, at the same time, relevant for cattle practice. Accurate prognosis not only allows for the early identification of patients requiring more intensive peri- and postoperative care but will also facilitate economic decision making in critical cases [17].

The objective of this retrospective study was to explore the suitability of a number of biochemical and clinical parameters to predict the fatality of the treatment outcome of cows with RDA or AV.

## 2. Materials and Methods

### 2.1. Animals

This manuscript presents the results of a retrospective study based on the caseload of a veterinary teaching hospital. Medical records from cows admitted to the Clinic for Cattle University of Veterinary Medicine, Hannover, Germany between 1 January 2015 and 30 November 2020 were considered for inclusion. The database of the teaching hospital was searched for records containing a diagnosis of either RDA or AV. Retrieved records were reviewed and included when the results of the complete physical examination and the standard hematological and blood biochemical analysis were available. For inclusion, patients furthermore needed to have undergone exploratory surgery during which the diagnosis of RDA or AV was confirmed.

### 2.2. Clinical Parameters

Parameters included in the analysis were age, stage of lactation, duration of hospitalization, as well as the following clinical parameters determined before surgery: Appetite, attitude, posture, rectal temperature (RT), hydration status, respiratory rate (RR), heart rate (HR), fecal output (volume, consistency, color, degree of fiber comminution), degree of rumen fill, stratification, and rumen motility (frequency and intensity). Hydration status was assessed by skin tent (upper eyelid tent) and eyeball recession. Visibility of the abomasum on the right flank, caudal to the last rib, and palpation of the dilated abomasum during rectal examination was also included in the analysis.

### 2.3. Laboratory Parameters

Blood samples collected from the jugular vein at the time of admission were processed as follows: blood collected in tubes containing EDTA as an anticoagulant was used for hematological analysis, which included leukocyte count (WBC), red blood cell count (RBC), thrombocyte count (PLT), mean corpuscular volume (MCV), mean corpuscular hemoglobin (MCH), mean corpuscular hemoglobin concentration (MCHC), hemoglobin concentration (Hb), and hematocrit (Hct). These analyses were conducted on an automatic hematology analyzer via the electrical impedance method (Celltac-α, MEK-6450, Nihon Kohden, Qinlab Diagnostik, Weichs, Germany). The Hct was determined via capillary centrifugation (Hettich Haematokrit centrifuge, Hettich Holding GmbH & Co. oHG, Kirchlengern, Germany).

Blood biochemical parameters were measured in serum obtained by centrifuging blood collected in serum separator tubes, which were kept in an upright position for 60 min before being centrifuged at 3000× *g* for 15 min at room temperature. The standard chemistry panel included the concentration of total protein (TP), albumin (Alb), total bilirubin, cholesterol (Chol), sodium (Na), potassium (K), chloride (Cl), inorganic phosphorus (Pi), total calcium (total Ca), magnesium (Mg), creatinine (Crea), urea, and selenium (Se), as well as activities in the serum of the enzymes aspartate aminotransferase (AST), gamma-glutamyl transferase (GGT), and glutamate dehydrogenase (GLDH). Biochemical analyses were conducted with a clinical chemistry analyzer (ABX Pentra^®^ 400, HORIBA Medical, Montpellier, France, or Cobas Mira^®^ Plus, Hoffmann La-Roche AG, Basel, Switzerland for Ca). Total protein was determined via refractometry.

Blood gas analyses were conducted on the blood collected in heparinized and calcium balanced capillaries (epoc Care-Fill™ Capillary Tube, Siemens Healthcare Diagnostics). This analysis included pH, partial pressure of carbon dioxide (pCO_2_) and partial pressure of oxygen (pO_2_), ionized calcium (Ca^2+^), L-lactate (Lact), and glucose (Gluc). Oxygen saturation (SO_2_), actual bicarbonate (HCO_3_^−^), base excess (BE), and anion gap (AG) were calculated using standard algorithms by the point of care unit. Measured values were corrected for the rectal temperature determined immediately prior to blood sampling. Blood gas analysis was performed on a point of care unit that was previously internally validated in our laboratory for use in cattle (EPOC Host and Reader, Siemens Healthineers AG, Erlangen, Germany).

Blood for hematology and biochemical analysis was kept at 6 °C until analysis, which occurred 4–18 h after sample collection. The blood gas analysis, as well as the determination of Hct and TP were performed within five minutes of blood sample collection.

### 2.4. Surgical Parameters

The following findings determined during exploratory surgery were included in the dataset: the grade of the abomasal dilatation, the volume of the abomasal fluid content, the amount of fluid content in the peritoneal cavity, and the presence of fibrin in the peritoneal cavity. In case of an AV, involvement of omasum and reticulum were recorded.

### 2.5. Statistical Analysis

Numerical data were tested for the normal distribution of residuals and homogeneity of variance using the Shapiro–Wilk test and by visually inspecting QQ plots. Cows were retrospectively classified as survivors and non-survivors. Non-survivors were patients with a confirmed diagnosis of RDA or AV that either died or were euthanized in the clinic; survivors left the clinic alive. Numerical data were analyzed as continuous variables and presented as mean ± SD stratified by survival status. These variables were furthermore analyzed as categorical variables. For this purpose, the value of each parameter was categorized for each patient as normal (within an established reference range) or as below or above the reference range for cattle. Categorical data are presented in contingency tables. Numerical variables were studied for differences between survivors and non-survivors using the student-*t*-test; categorical data underwent a frequency analysis. Differences between survivors and non-survivors were furthermore tested via logistic regression ana-lyses. Analyses using multivariate models were attempted to identify interactions, for example, between age or stage of lactation with different parameters. However, these wereabandoned as the relatively small number of non-survivors yielded insufficient statistical power for these approaches. A *p* < 0.05 was considered statistically significant. Statistical analysis was performed with a statistical software package (SAS 9.4; The SAS Institute, Cary, NC, USA).

## 3. Results

### 3.1. Animals

A total of 234 records of Holstein-Friesian cows with a diagnosis of RDA or AV obtained from the database search were included in the dataset of this study. The mean age of the cows included was 4.5 ± 1.7 years. A total of 226 cows were lactating, of which 96 were between 1 and 15 days in milk (DIM), and 73 cows were between 16 and 43 DIM. Only eight animals were dry at the time of admission.

Of the 234 cows, 193 (82.5%) were discharged from the clinic (survivors), and 41 (17.5%) animals died or were euthanized (non-survivors). The mean hospitalization time of the survivors was 8 ± 5 days. From the non-survivors, 13 cows died (n = 4) or were euthanized (n = 9) during surgery. The remaining 28 non-survivors died or were euthanized within 7 ± 5 days post admission. The most common reasons for euthanasia during the surgery were the presence of a perforated abomasal ulcer, feed particles in the abdominal cavity, and peritonitis (n = 8).

### 3.2. Concurrent Diseases

The most common concurrent conditions diagnosed at admission in survivors were: ketosis (urine acetoacetate > 2.5 mmol/L; [18]) (n = 61), mild clinical mastitis (score 1) (n = 31; [19]), metritis grade I or II (n = 10; [20]), udder cleft dermatitis (n = 9), and severe clinical mastitis (score 3) (n = 2; [19]). The most frequent concurrent conditions diagnosed at admission in non-survivors were mild clinical mastitis (n = 6) and ketosis (n = 4). Melena, defined as the presence of black feces, was diagnosed in 10 survivors and in 4 non-survivors.

### 3.3. Clinical Findings

The clinical variables that differed significantly between survivors and non-survivors are summarized in Table 1. Rectal temperature did not differ between groups in absolute terms, but values above or below the reference range were more frequently determined in non-survivors than in survivors (Table 1). Furthermore, non-survivors had higher mean RR (*p* < 0.01) and mean HR (*p* < 0.0001) when compared with survivors. Non-survivors were diagnosed more frequently with tachypnea (*p* = 0.02) and tachycardia (*p* = 0.0001) than survivors (Table 1).

Non-survivors were diagnosed with a dull or obtunded demeanor more frequently than survivors (41.4% vs. 14%, respectively, *p* < 0.0001), had moderate or poor appetite more often, and were found to be moderately or severely dehydrated more frequently, based on both skin tent duration and eyeball recession when compared with survivors (Table 1). Variables characterizing rumen fill and motility (rumen fill, fiber mat, motility) were more often reduced in non-survivors than in survivors. In non-survivors, the dislocated abomasum was more often visible on the right flank, caudal to the last rib, and was accessible more frequently during a rectal examination than in survivors (Table 1).

The stage of lactation, as well as variables related to defecation (amount of fecal output, fecal consistency, fecal color, and degree of comminution of plant fragments in feces) did not differ between survivors and non-survivors (*p* > 0.05).

### 3.4. Laboratory Findings

The hematological and blood biochemical parameters are summarized in Table 2 and Table 3. The variables glucose, L-lactate, Na, Cl, Crea, WBC, and Pi differed significantly between survivors and non-survivors in numerical and categorical comparisons but not based on logistic regression analysis. Non-survivors had a higher mean WBC and were leukocytotic more frequently than survivors (Table 2 and Table 3). Serum urea was the only blood biochemical variable with statistically significant differences between survivors and non-survivors in all statistical analyses. Serum Na, Cl, and Ca^+2^ concentrations were lower, and Pi and Mg were higher in non-survivors than survivors (Table 2). For K and total Ca, differences between groups were identified during categorical analysis, with a higher frequency of hypokalemia and hypocalcemia in non-survivors than in survivors (Table 3). Logistic regression analysis yielded higher odds ratios for non-survivors to be hypocalcemic (total Ca) and uremic and lower odds ratios to be hyperbilirubinemic when compared with survivors (Table 4).

The pO_2_ and SO_2_ in venous blood were lower, and the Lact and the AG were higher in non-survivors when compared with survivors (Table 2). No differences between groups were found for MCH, MCHC, MCV, PLT, Alb, Chol, TP, Se, GGT, GLDH, pH, pCO_2_, BE, and HCO_3_^−^.

### 3.5. Surgical Findings

None of the cows had a laparotomy performed prior to admission. Four animals had a history of unsuccessful attempts of minimally invasive fixation of the abomasum immediately prior to referral. All of the cows underwent a right flank laparotomy and omentopexy within hours of admission. A dose of 0.5 mg/kg of Meloxicam (Animeloxan^®^, Animedica GmbH, Bösensell, Germany) was administered subcutaneously as a standard procedure before surgery in each case. Parenteral fluid therapy was administered intravenously via drip infusion (10–20 L 0.9% NaCl solution in most cases supplemented with KCl to obtain a concentration of K of 30 mmol/L) before, during, or after the surgery. Oral rehydration via administration of 30 L of water containing either 100–150 g KCl or 150 g NaCl with an orogastric tube was performed in many cases after surgery. The type and duration of the fluid therapy was at the surgeon’s discretion.

Local anesthesia was obtained using a combination of distal paravertebral block and infiltration anesthesia of the incision line with procaine hydrochloride (20 mg/mL) and epinephrine (0.025 mg/mL) (Isocain^®^ 2%; Selectavet Dr. Otto Fischer, Weyarn-Holzolling, Germany).

**Figure 1:**
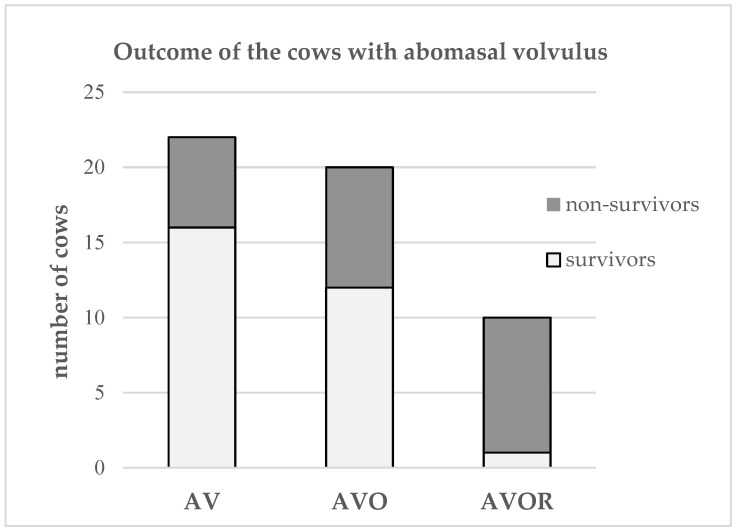
Classification of the 52 cows with volvulus of the abomasum according to surgical findings and the outcome (survivors or non-survivors). In 22 cases, only the abomasum was twisted (AV), in 20 cases the omasum was also included in the volvulus (AVO), and in 10 cases the omasum and the reticulum were also twisted (AVOR).

Abomasal displacement to the right was diagnosed in 182 cases, and AV was found in 52 cases. In the cases of AV, only the abomasum was twisted in 22 cases, both the abomasum and omasum were twisted in 20 instances (AVO), and the abomasum, omasum and reticulum were twisted in 10 cases (AVOR). Abomasal volvulus was diagnosed in only 15% of survivors but over 43% of non-survivors (Table 1). The prognosis of survival decreased even further in cows with AV when the omasum (AVO) or omasum and reticulum (AVOR) were also twisted (Figure 1).

The abomasum was repositioned and fixated on the right flank via omentopexy. An abomasotomy with fluid decompression (approximately 15–40 Liters) was performed in five cows with AVO and in two cows with AVOR. Out of all abomasotomy cases (n = 7), only two cows survived and were discharged from the clinic.

Surgery was performed with the cow standing in 226 cases and on the recumbent cow in eight cases. None of the cows operated in sternal or lateral/sternal recumbency survived (Table 1). Local intraabdominal adhesions were identified in 16 cases.

The abomasum was more frequently moderately to severely dilated and filled with fluid in non-survivors than in survivors. Increased amounts of fluid in the peritoneal cavity was also diagnosed more frequently in non-survivors than in survivors. The frequency with which fibrin in the abdominal cavity was found did not differ between groups. The abomasal wall was found to be markedly discolored in 8 of 41 (19.5%) non-survivors but only in 1 survivor (0.5%). Generalized peritonitis as a result of a perforating abomasal ulcer was the most common necropsy finding in four out of nine cows that underwent necropsy postoperatively.

## 4. Discussion

The objective of this retrospective study was to identify predictors of survival in cows diagnosed with RDA or AV among preoperative clinical and laboratory findings obtained immediately prior to surgery, as well as intraoperative findings.

Of the preoperative clinical parameters, the strongest predictive values were identified for tachycardia and clinical dehydration. Tachycardia was associated with poorer prognosis in cows diagnosed with RDA or AV in earlier studies [7,8,10,12]. Stress (sympathetic nervous system stimulation) and cardiovascular disturbances such as hypovolemia, compression of the caudal vena cava resulting from severe abomasal distention, and volvulus were likely contributing factors to tachycardia [7,21].

Marked dehydration and hemoconcentration were more frequent in non-survivors than survivors, and this can be considered a consequence of extensive fluid sequestration in the displaced abomasum and the inability to transfer fluid to the duodenum [22,23]. This interpretation is corroborated with the higher incidence of a markedly dilated abomasum in non-survivors (Table 1). In this context, the negative postoperative outcome of all eight cows that underwent surgery in a recumbent position was probably associated with a more pronounced fluid sequestration and ensuing cardiovascular and metabolic disruption. This assumption is supported by the lower Cl that was found in non-survivors than in survivors, which is known to occur as a result of a more pronounced sequestration of abomasal fluid and the inhibition of chloride reabsorption in the duodenum [3,24].

The prognostic value of the volume of abomasal fluid content was previously reported [6,16]. Prolonged distention in combination with strangulation in cases of AV are thought to cause irreversible neuromuscular damage resulting in functional disturbances of the abomasum [6,16,25]. In our study, we had similar results, as only one out of nine cases with remarkably discolored (bluish) abomasal serosa returned to the farm. Discoloration of the abomasal wall as reported here is presumably the result of impaired vascular perfusion and was also found to be related to worse prognosis in this and earlier studies [8,9,10].

A more pronounced distension of the displaced abomasum in non-survivors compared to survivors presumably also explains why the displaced abomasum was palpable rectally and was visible from the right side, caudal to the last rib, more frequently in non-survivors compared to survivors. Similar findings were previously reported [5,8]. One earlier study, however, found no difference in productive outcome based on the width of the positive percussion auscultation area or the rectally palpable abomasum [7].

In this context, cases requiring abomasotomy during surgery for the decompression and correction of the abomasal twist are considered as having a poorer prognosis because of prolonged and severe tissue damage in combination with the loss of electrolytes in the trapped abomasal fluid [10]. This is in agreement with the results of the present study in which only two out of seven cows undergoing abomasotomy and fluid decompression survived.

The poorer prognosis of cows with AV reported in the present study is consistent with several earlier reports [2,10]. Similarly, the poorer outcome in cows diagnosed with AVO and AVOR found in our study (Figure 1) is in agreement with earlier findings [8,16,25,26].

The assessment of some clinical parameters or surgical findings may be subjective, with individual variations depending on the clinician. However, all patients included in this study underwent the same clinical exam preoperatively using standardized clinical procedures conducted by experienced clinic assistants who also performed the surgery.

Of the blood and serum biochemical parameters included in this study, serum urea had the strongest predictive value for non-survival. Azotemia in cows with abomasal displacement has been attributed to dehydration and ensuing enhanced tubular water reabsorption in the kidneys [22,27]. This interpretation is corroborated by the higher Crea levels in non-survivors when compared with survivors, as well as higher Hct, Hb, and RBC values in non-survivors compared with survivors. Urea was also found to be a better predictor of a postoperative outcome when compared with Crea in calves, according to a recent study [28]. Serum urea is considered to be more sensitive for the diagnosis of dehydration, as elevated values are the result of increased renal tubular reabsorption and decreased renal glomerular filtration, while elevated Crea are only the result of decreased renal glomerular filtration [7].

Hemoconcentration with increased RBC, Hb, and Hct were also reported in earlier studies on cows with abomasal displacement [22]. The underlying cause of leukocytosis could not be determined, as differential leukograms were not available. Increased WBC could be the result of an inflammatory response, dehydration, elevated cortisol levels, or a combination of several of these factors [29]. In a previous study including 106 cows with RDA or AV, the productive outcome of cows was not associated with WBC [8], while another study found leukocytosis to be associated with poor prognosis in cows with RDA [30].

Higher Lact values and the higher frequency of hyperlactatemia (L-lactate) in non-survivors is most likely related to reduced abomasal perfusion and ensuing tissue hypoxia [31]. The use of Lact as an outcome predictor for cows with RDA or AV was suggested earlier [13]. The authors of that study proposed a combination of Lact with HR for a more accurate outcome prediction [13]. However, according to a more recent report, measuring Lact six hours after initiating surgery was found to have a better predictive value for a negative outcome in cows with acute abdominal disorders when compared to a single preoperative measurement [15].

Despite more frequent and more pronounced hyperlactatemia in non-survivors, the BE was not significantly different between survivors and non-survivors in this study. Similar findings were previously reported [7]. A possible explanation for this apparently paradoxical finding could be the more pronounced occurrence of metabolic alkalosis that is commonly observed in animals with high gastrointestinal tract obstruction and in non-survivors [6,7]. In an older study, a trend *(p* = 0.08) towards a lower survival rate was reported when the BE ≤ −0.1 mEq/L [32].

Hyperglycemia, as in this study, was also found to be a predictor of a poor outcome in a previous report [30]; the hyperglycemia in non-survivors was presumably the result of stress [33].

A significantly lower serum Na concentration in non-survivors than survivors suggests a tendency toward hypotonic dehydration in non-survivors. The sequestration of Na in the abomasum is an implausible underlying mechanism, because the Na concentration in abomasal fluid in the range of 40 mmol/L is well below the concentration in plasma and is significantly lower in cows with abomasal displacement than in healthy cows [24]. A more plausible mechanism is pseudohyponatremia driven by marked hyperglycemia in non-surviving animals. Excess Gluc in the extracellular space causes water to move toward the extracellular space, thereby diluting the concentration of predominantly extracellular electrolytes such as Na and Cl [34]. Hyperglycemia is likely to have contributed to the difference in serum Cl and K through this mechanism. More pronounced hypochloremia in non-survivors than in survivors is presumably attributable to a more pronounced sequestration of abomasal fluid and consequently greater impairment of Cl reabsorption from the duodenum in non-survivors [3,24]. Hypokalemia, which is a common finding in RDA or AV, has been attributed to feed intake depression, sequestration of K in the abomasum, and alkalemia, resulting in a compartmental shift of K from the extracellular to the intracellular space [7,11,35]. In our study, the numerical analysis of K did not reveal outcome related differences, whereas categorical analysis revealed that hypo- and hyperkalemia were more frequent in non-survivors than in survivors (Table 2). We assume that in cases with more pronounced dehydration, the metabolic alkalosis, typically encountered in cows with abomasal displacement and effects of feed intake depression, may be antagonized by an impaired glomerular filtration rate and lactate accumulation.

Inorganic phosphorus and Mg, which also showed higher values in non-survivors than in survivors in this study, were also reported to increase in cows with RDA or AV due to dehydration-decreased renal perfusion [27]. Similar findings were reported in a recent study, where higher values of Mg and Pi in cows with RDA were associated with a poor prognosis [30].

In this study, the concentration of Ca^2+^ (but not total Ca) was found to be lower in non-survivors than survivors. A similar effect was apparent in the categorical analysis and in the logistic regression analysis for total Ca. Lower total Ca concentrations in cows with abomasal displacements in comparison to healthy cows were previously reported, and have been discussed as a causative factor rather than a consequence of abomasal displacement [36,37]. Assuming that the level of hypocalcemia contributes to a reduction in abomasal motility, low total Ca levels may contribute to the increased risk of a fatal outcome. Lower levels of total Ca could, however, also be associated with leukocytosis and an upregulated inflammatory status that would require additional Ca for an oxidative burst [38]. In earlier studies, total Ca concentration was not associated with a therapeutic outcome in cows with RDA, AV [7,8,10], or LDA [12,22]. To the best of our knowledge, this is the first study reporting that calcium is associated with an increased risk of non-survival in cows with RDA. A factor that may have contributed to the better predictive value of Ca^2+^ when compared with total Ca is the binding of Ca^2+^ to anions such as lactate and inorganic phosphate [39]. The more frequent occurrence of hyperlactatemia and hyperphosphatemia in combination with higher plasma phosphate and Lact concentrations found in non-survivors in this study may have contributed to more pronounced hypocalcemia (based on Ca^2+^) in non-survivors compared to survivors.

Of all the cows included in this retrospective study, 82.5% (193/234) were discharged from the clinic (survivors). A similar survival rate (86.2%; 362/420) was reported in a previous study of cows with RDA or AV [40]. Survival rates vary considerably between reports, which may at least be partially attributable to differing definitions of positive and negative outcomes [8,41]. A limitation of this study is the definition of survival and non-survival, which is only based on whether a patient left the teaching hospital alive or not and thus, neither took into consideration long-term survival nor productivity after treatment. Furthermore, in our study, we did not subdivide cows with RDA and AV, primarily because the number of cases with AV would not have provided sufficient statistical power for a meaningful analysis. Limited statistical power resulting from the relatively small number of non-survivors was also what precluded a meaningful and more in-depth statistical analysis using multivariate analyses exploring the prognostic value of specific parameters, for example, by age or stage of lactation. This study also only included preoperative blood biochemical analyses, as perioperative treatment regularly included non-standardized parenteral and oral fluid therapy, which was likely to have considerably altered blood biochemical parameters. The predictive value of β-hydroxybutyrate and nonesterified fatty acids in the blood of cows with LDA was previously reported [42]. However, these parameters were, unfortunately, not available in the retrospective dataset of this study. In general, this report presents the typical weaknesses of a retrospective data analysis such as variation and lack of control of the quality of data collection and recording. This may be an issue in particular for medical emergencies, such as when patients with suspected AV are admitted at any time of the day or the night. In such cases, the priority of attending veterinarians is timely and efficient medical treatment rather than the meticulous recording of clinical findings. Furthermore, the data analysis of a retrospective dataset can evidently only refer to available data rather than actively select parameters that may be of interest for the research question under investigation. The results reported here do, however, provide worthwhile information to be considered in future prospective studies.

In a recent retrospective study, cows with RDA and AV had a lower mean pH and higher specific gravity in urine than cows with LDA [5]. Moreover, marked hematuria and glucosuria was a more frequent finding in cows with AV than in cows with LDA. Further studies investigating the role of urine parameters for the prediction of survival outcomes in cows with RDA and AV would be of great interest.

## 5. Conclusions

Based on the results presented here, the best predictive value for the outcome of cows after the surgical correction of an abomasal displacement to the right or abomasal volvulus was determined to be serum urea, followed by creatinine, sodium, inorganic phosphorus, and L-lactate. Heart rate, clinical hydration, and standing ability were the most important clinical predictors for a postoperative outcome. Abomasal dilatation and abomasal volvulus were the most important surgical indicators for a worse prognosis.

## Figures and Tables

**Table 1:** Preoperative clinical and surgical findings, which were significantly different between survivors and non-survivors (frequency analysis).

**Clinical Finding**	**Survivors**		**Non-Survivors**		**Chi-Square**
	**n**	**(%)**	**n**	**(%)**	***p* Value**
**Respiratory rate**	192		41		0.02
<16 bpm		0		0	
16–28 bpm		63.5		43.9	
>28 bpm		36.5		56.1	
**Heart rate**	191		39		0.0001
<60 bpm		2.6		0	
60–80 bpm		62.3		28.2	
>80 bpm		35.1		71.8	
**Rectal Temperature**	192		41		0.0001
<38.3 °C		23.4		39	
38.3–39.5 °C		69.8		43.9	
>39.5 °C		6.7		17.1	
**Feed intake**	192		41		0.002
good		0.5		0	
moderate		16.2		9.8	
poor		40.1		14.6	
no interest		43.2		75.6	
**Attitude**	191		41		<0.0001
normal (alert, bright)		75		51.2	
slightly dull		11		14.6	
obtunded		3		26.8	
nervous		0.1		7.3	
**Rumen fill**	192		41		0.005
good		24.5		24.4	
moderate		67.7		48.8	
poor		7.8		26.8	
Fiber mat	192		41		0.01
good		31		19.5	
moderate		51		39	
poor		18		41.5	
**Rumen motility (contraction frequency/intensity)**	191		40		0.0009
3		1.1		0	
2		33.5		20	
1		55		45	
0		10.5		35	
**Abomasum visible (caudal to the last rib)**	192		41		0.0003
no		77		43.9	
yes		23		56.1	
**Abomasum accessible at rectal examination**	190		41		0.001
no		64.3		34.1	
yes		35.7		65.9	
**Eyeball recession**	191		26		<0.0001
normal		78		38.4	
slightly sunken		13.6		15.3	
moderately sunken		6.8		34.6	
severely sunken		1.6		11.5	
**Skin tent**	191		41		<0.0001
good		36.1		12.2	
moderately decreased		54.5		24.4	
severely decreased		9.4		63.4	
**Standing ability for surgery**	193		41		<0.0001
yes		100		80.5	
no		0		19.5	
**Surgical Finding**	**Survivors**		**Non-Survivors**		**Chi-Square**
	**n**	**(%)**	**n**	**(%)**	***p* Value**
**Abomasal dilatation**	193		41		<0.0001
slightly distended		60.9		7.3	
moderately distended		21.5		34.2	
markedly distended		17.6		58.5	
**Abomasal volume**	192		41		0.0004
normal or slightly increased		81.9		53.6	
moderately increased		9.8		22	
markedly increased		8.3		24.4	
**Abomasal volvulus**	193		41		<0.0001
no		85		56.1	
yes		15		43.9	
**Fluid content in the peritoneal cavity**	193		41		0.002
normal		68.9		48.8	
slightly increased		20.2		19.5	
markedly increased		10.9		31.7	

**Table 2:** Preoperative laboratory parameters in survivors and non-survivors (*t*-test). The parameters, which were significantly different between groups (*p* < 0.05), are presented in **bold**.

Variable	Unit	Survivors			Non-Survivors			*p* Value
		n	Mean	SD	n	Mean	SD	
WBC	/μL	184	9617	4689	38	14184	8015	**<0.0001**
RBC	10^6^/μL	184	7.0	1.2	38	7.7	1.5	**0.003**
PLT	cells/μL	176	431,000	162,000	31	385,000	135,000	0.14
MCV	μm^3^	184	51.6	4.3	38	52.3	6.0	0.46
MCH	pg	184	15.1	13	38	15.2	1.6	0.83
MCHC	g/dL	184	29.3	1.4	38	29.1	1.24	0.47
Hb	g/dL	184	10.6	1.6	38	11.6	2.0	**0.0009**
Hct	%	184	36.0	5.6	38	39.7	6.4	**0.0004**
TP	g/L	184	67	8.8	38	67.3	10.7	0.80
Alb	g/L	184	30.5	3.7	38	29.8	4.5	0.31
Bili	μmol/L	183	13.9	7.5	37	12.1	7.9	0.20
Chol	mmol/L	184	2.2	1.1	38	2.26	1.06	0.71
Na	mmol/L	184	137.0	4.2	38	133.5	4.3	**<0.0001**
K	mmol/L	183	4.07	0.71	38	4.07	1.04	0.97
Cl	mmol/L	184	91.0	9.9	38	82.5	10.0	**<0.0001**
Pi	mmol/L	185	1.7	0.6	38	2.3	0.8	**<0.0001**
Total Ca	mmol/L	185	2.13	0.29	38	2.22	0.23	0.06
Mg	mmol/L	185	0.8	0.2	38	1.1	0.3	**<0.0001**
Crea	μmol/L	184	91.0	36.1	38	157.5	107.9	**<0.0001**
Urea	mmol/L	184	8.0	5.5	38	15.9	8.7	**<0.0001**
Se	μg/L	178	96.5	21.3	38	92.3	28.1	0.29
AST	U/I	184	227	190	38	290.7	258	0.06
GGT	U/I	184	83.2	61	38	84.9	47.1	0.42
GLDH	U/I	184	131	139	38	141	117	0.35
pH		120	7.42	0.06	33	7.42	0.07	0.82
pO_2_	mm Hg	120	36.6	9.3	33	30.2	8.5	**0.0005**
pCO_2_	mm Hg	120	30.7	6.8	33	31.7	6.1	0.45
Ca^2+^	mmol/L	119	1.03	0.14	33	0.97	0.14	**0.04**
Lact	mmol/L	120	3.9	3.1	33	8.1	3.7	**<0.0001**
Gluc	mg/dL	120	161.1	94.6	33	221.7	86.0	**0.001**
SO_2_	%	120	62.2	13.7	33	49.1	18.0	**<0.0001**
HCO_3_^−^	mmol/L	120	29.6	6.6	33	30.4	6.0	0.55
BE	mmol/L	120	5.3	7.4	33	6.2	6.9	0.53
AG	mmol/L	114	20.7	5.6	31	24.1	6.1	**0.0003**

SD: standard deviation; WBC: white blood cell count; RBC: red blood cell count; PLT: thrombocyte count; MCV: mean corpuscular volume; MCH: mean corpuscular hemoglobin; MCHC: mean corpuscular hemoglobin concentration; Hb: hemoglobin; Hct: hematocrit; TP: total protein; Alb: albumin; Bili: total bilirubin; Chol: cholesterol; Na: sodium; K: potassium; Cl: chloride; Pi: inorganic phosphorus; total Ca: total Calcium; Mg: magnesium; Crea: creatinine; Se: selenium; AST: aspartate aminotransferase; GGT: gamma-glutamyl transferase; GLDH: glutamate dehydrogenase; pO_2_: partial pressure of oxygen; pCO_2_: partial pressure of carbon dioxide; Ca^2+^: ionized calcium; Lact: L-lactate; Gluc: glucose; SO_2_: oxygen saturation; BE: base excess; HCO_3_^−^: actual bicarbonate; AG: anion gap.

**Table 3:** The difference of the proportion of animals with values below or above the reference range between survivors and non-survivors for the blood biochemical parameters studied.

Variable	Unit	Reference Range		n (Total)	Below Reference Range		Above Reference Range		Chi-Square
					Survivors (%)	Non- Survivors (%)	Survivors (%)	Non- Survivors (%)	*p* Value
Gluc	mg/dL	74	100	153	5	0	74.2	97	0.02
Total Ca	mmol/L	2.1	3	223	15.8	36.2	0	0	0.01
Ca^2+^	mmol/L	1.15	1.33	119	79.8	87.9	0.8	0	0.54
Cl	mmol/L	90	110	222	37.5	73.7	0.5	0	0.0002
Crea	μmol/L	>150		222	95.1	60.5	4.9	39.5	<0.0001
Urea	mmol/L	<8		222	67.4	21	32.6	79	0.0001
WBC	/μL	8000	10,000	222	40.2	21	38.5	57.9	0.03
Na	mmol/L	135	145	222	22.3	63.2	2.2	0	<0.0001
Bili	μmol/L	>7		220	18	32.4	82	67.6	0.05
Lact	mmol/L	<1.39		153	61.7	12.1	38.3	87.9	<0.0001
K	mmol/L	3.5	4.5	221	19.7	31.6	20.8	31.6	0.04
Pi	mmol/L	1.1	2.4	223	10.8	7.9	9.2	42.1	<0.0001

Gluc: glucose; Total Ca: total calcium; Ca^2+^: ionized calcium; Cl: chloride; Crea: creatinine; WBC: white blood cell count; Na: sodium; Bili: total bilirubin; Lact: L-lactate; K: potassium; Pi: inorganic phosphorus.

**Table 4:** Blood biochemical parameters with odds ratios for non-survivors compared to survivors that were significantly different from 1 (logistic regression analysis).

Variable	Odds Ratio	95% Wald Confidence Interval	*p* Value
Low Calcium (<2.1 mmol/L)	3.05	1.03–9.02	0.04
High Serum Urea (>8 mmol/L)	4.74	1.25–17.93	0.02
High Bilirubin (>7.0 μmol/L)	0.34	0.12–0.92	0.03

Calcium: total calcium.

## Data Availability

The data analyzed and presented in this study are available upon reasonable request from the corresponding author. The data are not publicly available due to the protection of the data privacy of the patients of the Clinic for Cattle, University of Veterinary Medicine Hannover, Foundation, Hannover, Germany.
